# The mathematics of market timing

**DOI:** 10.1371/journal.pone.0200561

**Published:** 2018-07-18

**Authors:** Guy Metcalfe

**Affiliations:** School of Mathematical Sciences, Monash University, Clayton, VIC, Australia; Universidad Veracruzana, MEXICO

## Abstract

Market timing is an investment technique that tries to continuously switch investment into assets forecast to have better returns. What is the likelihood of having a successful market timing strategy? With an emphasis on modeling simplicity, I calculate the feasible set of market timing portfolios using index mutual fund data for perfectly timed (by hindsight) all or nothing quarterly switching between two asset classes, US stocks and bonds over the time period 1993–2017. The historical optimal timing path of switches is shown to be indistinguishable from a random sequence. The key result is that the probability distribution function of market timing returns is asymmetric, that the highest probability outcome for market timing is a below median return. Put another way, simple math says market timing is more likely to lose than to win—even before accounting for costs. The median of the market timing return probability distribution can be directly calculated as a weighted average of the returns of the model assets with the weights given by the fraction of time each asset has a higher return than the other. For the time period of the data the median return was close to, but not identical with, the return of a static 60:40 stock:bond portfolio. These results are illustrated through Monte Carlo sampling of timing paths within the feasible set and by the observed return paths of several market timing mutual funds.

## Introduction

Market timing is an investment technique whereby an investment manager (professional or individual) attempts to anticipate the price movement of asset classes of securities, such as stocks and bonds, and to switch investment money away from assets with lower anticipated returns into assets with higher anticipated returns. Market timing managers use economic or other data to calculate propitious times to switch. Market timing seems a popular approach to investment management, with Morningstar listing several hundred funds in its tactical asset allocation (TAA) category—TAA being an industry name for market timing—and mainstream fund managers advertising their ability to switch to defensive assets when stock markets seem poised for a downturn. The antithesis of market timing, and another broadly popular investing approach, is buy-and-hold, whereby investment managers allocate static fractions of their monies to the available asset classes and then ignore market price gyrations.

Is market timing likely to be successful relative to investing in a static allocation to the available asset classes? The literature in this area is focused on developing sophisticated statistical tools that can detect and measure the market timing ability of professional fund managers [1]. Numerous uses of these techniques over decades have produced mixed results [2–7]. Some authors detect no market timing ability, while others report statistically significant evidence of market timing ability. On the other hand, Dalbar measures the market timing results of the average individual investor through mutual fund sales, redemptions and exchanges [8]. These studies find unambiguously that market timing by the average investor is unsuccessful relative to a static allocation. The ambiguous results for successful market timing from professional managers suggests that, at minimum, it is difficult to market time successfully, while the unambiguous results for individuals strongly suggests that it is easy to market time unsuccessfully.

My goal here is both different and simpler than statistical tests to detect market timing. I want to create a simple model to ask the question, what is the likelihood of successful market timing? Or more precisely, what is the return probability distribution function (PDF) for market timing? Is the PDF of market timing returns symmetric? If it is hard to obtain above average returns by market timing, is it also hard to obtain below average returns? What is the most basic mathematics of market timing?

I try in this paper to evoke a similar spirit to Sharpe’s “The Arithmetic of Active Management” [9], in which elementary arithmetic is all that is required to demonstrate why active management must in aggregate under perform low-cost index funds. While I will need to invoke elementary probability theory, it will show that the most probable outcome of market timing is to under perform a buy-and-hold, suitably weighted average of the available asset classes. Moreover, as I build the simple model from the returns of US stock and bond total market index funds since 1993, market returns over that time period mean that the suitably weighted average portfolio, while not identical with the 60:40 stock:bond balanced fund, is in practice barely distinguishable from it.

In the rest of the paper my approach will be to calculate the boundaries of the feasible set of market timing portfolios using fund data for perfectly timed (by hindsight) switching between two asset classes, stocks and bonds. From this analysis I also obtain the historically optimal timing path of switches, which the NIST (National Institute of Standards and Technology, U.S. Department of Commerce, www.nist.gov) suite of tests for randomness shows is indistinguishable from a random sequence. The key elementary result is that the geometric mean of market timing returns has an asymmetric PDF. One implication of this is that the most probable market timing return is below the median return, which can be directly calculated to be given by a static portfolio weighted by the relative fraction of time periods that each asset class outperforms the other. These results are illustrated through Monte Carlo sampling of timing paths within the feasible set and by the return paths of several market timing funds with comparably long, publicly available data. To begin, in the next section I describe the data.

## Data

**Fig 1:**
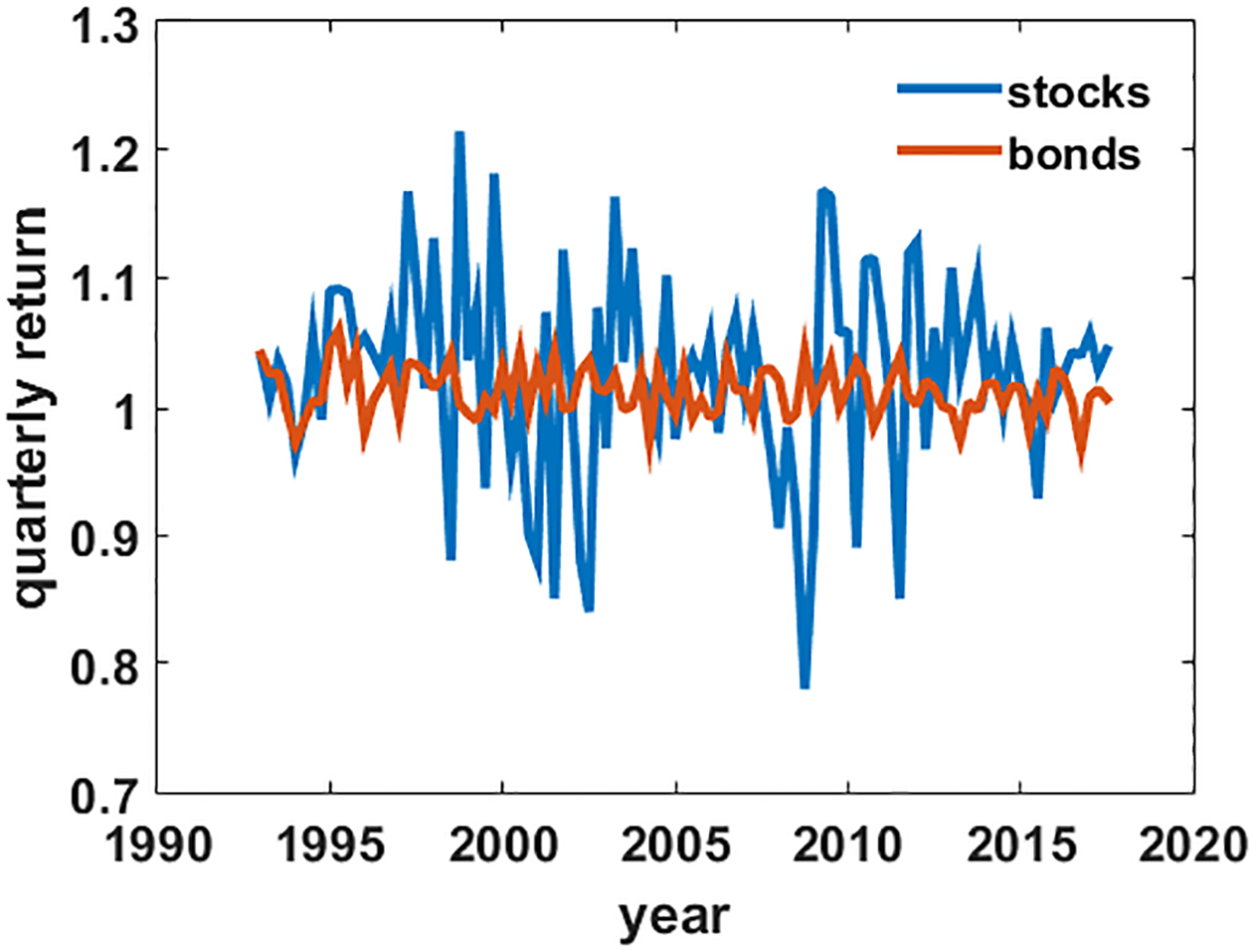
Quarterly returns data. Quarterly return time series for stock and bond total market index funds, 1993–2017. Returns are in multiplicative form.

The data consists of time series of quarterly returns for three index funds starting in 1993, the advent of the youngest of the three funds, and ending in Q3 2017. The series covers 24 years, and there are *N* = 99 data points per series. The funds, all from Vanguard, are Total Stock Market, Total Bond Market, and Balanced Index, the last a static portfolio of 60% Total Stock and 40% Total Bond. Other information on these funds is in S1 Appendix. Fig 1 shows the quarterly return time series for stocks and bonds. Because the data are from live funds, calculated return paths are net of management and trading costs; however, tax consequences are ignored. For quarterly switching taxes would likely be substantial, but the effect would only dampen the spread of net returns and change only the quantitative, not the qualitative results of the model. Note that because fund data are the basic building blocks of the model, all return paths calculated could have been obtained by an investor during the time period.

Since the way to calculate total return is to multiply the sub-period returns together, I trivially transform the original data to multiplicative form, e.g. a +3% return becomes 1.03 and a −3% return becomes 0.97. The differences between multiplicative and additive random processes will be important in the subsequent analysis.

## Two asset, all or nothing market timing model

Here I define the simple two asset market timing model with all or nothing quarterly switches, emphasizing the deliberate choice to assume a simple model in order to gain insight into the fundamental mathematics. Using perfect hindsight, it is easy to identify the best and worst possible market timing portfolios, which form the boundaries of the feasible return paths for all market timing portfolios, i.e. all possible market timing portfolios lie between the boundaries of the feasible set. (Technically it is all market timing portfolios that conform to the assumptions of the model; however, in the discussion section we will see that real, non-conforming market timing funds fall within the feasible set.) I reveal the optimal (highest possible return) timing sequence and test it for randomness. A later section focuses on deriving the return PDF for the model.

### Model

The model consists of quarterly all or nothing switches between stocks and bonds. In the *i*th time period *t*_*i*_ the return of stocks is denoted *r*_*si*_ and the return of bonds is denoted *r*_*bi*_. A *timing path* is the binary sequence *f*_*i*_ that is

$$\begin{array}{c} f_{i}=\left\{ \begin{array}{cc} 1 & \;\text{if}\;\text{during}\;t_{i}\;r_{si}>r_{bi}\\ 0 & \;\text{if}\;\text{during}\;t_{i}\;r_{si}<r_{bi}. \end{array}\right. \end{array}$$ (1)

In other words *f* is set to *f* = 1 when the stock return is larger than the bond return and set to *f* = 0 when the bond return is larger than the stock return. A special class of timing path has *f* = constant and is termed a static allocation or buy-and-hold portfolio. I call a *return path*, denoted *ρ*, the sequence of returns generated by a particular timing path *f*_*i*_. The *j*th return path is given by

$$\begin{array}{c} \rho_{j}=\prod_{i}^{N}\left(f_{ij}r_{si}+\left(1-f_{ij}\right)r_{bi}\right). \end{array}$$ (2)

The geometric mean of a return path is given by $\rho_{j}^{1/N}$.

### Feasible set

**Fig 2:**
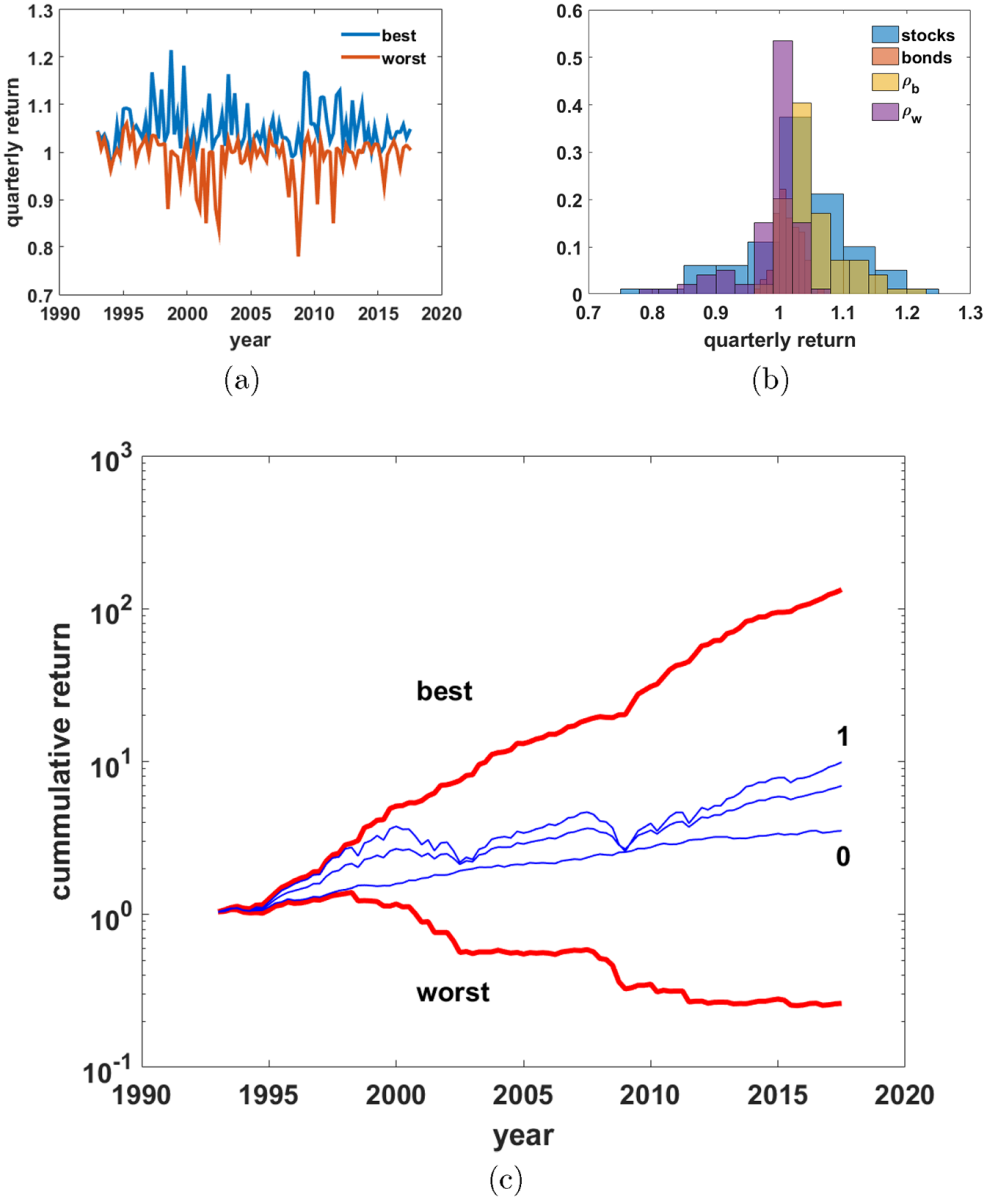
Two asset, all or nothing market timing model. Two asset, all or nothing market timing model switches to whichever of the two assets classes will have the better return that quarter. (a) Quarterly returns of the best and worst market timing portfolios as a function of time in multiplicative form. (b) Histograms of returns for the indicated data sets. (c) Feasibility envelope plotted on semi-log axes. Thick red lines are the best and worst possible return paths over this time period. Blue lines are the three data sets: stocks (*f* = 1), bonds (*f* = 0), and balanced (*f* = 0.6). The fixed portfolio lines order as expected from *f* = 0 to *f* = 1.

With perfect hindsight the best and worst performing return paths are easily found. In the notation of Matlab code Eq (2) becomes for the best *ρ*_*b*_ and worst *ρ*_*w*_ possible return paths

$$\begin{array}{c} \rho_{b}=\text{cumprod(max(stocks,}\;\text{bonds))} \end{array}$$ (3a)

$$\begin{array}{c} \rho_{w}=\text{cumprod(min(stocks,}\;\text{bonds))}, \end{array}$$ (3b)

and the timing path for *ρ*_*b*_ is given by *f*_*b*_ = (stocks > bonds); similarly for *ρ*_*w*_. Fig 2(a) shows the quarterly return series for *ρ*_*b*_ and *ρ*_*w*_, while Fig 2(b) shows histograms of quarterly returns for stocks, bonds, *ρ*_*b*_, and *ρ*_*w*_. There are no surprises: partitioning returns by Eq (3) puts the positive return, right tail of the stocks distribution into *ρ*_*b*_, while excluding the negative return, left tail. The reverse happens to *ρ*_*w*_.

Fig 2(c) plots several return paths on semi-log axes. The best and worst possible return paths for this period are thick red lines. Blue lines are the fund data for stocks (*f* = 1), bonds (*f* = 0) and balanced (*f* = 0.6). The returns of the fixed portfolios are ordered as expected with *f* = 0 producing the lowest returns of the fixed *f* portfolios and *f* = 1 producing the highest. Note, however, that the large difference in returns normally associated with stocks and bonds is dwarfed by the difference in returns between the best and worst market timing portfolios. The potential reward to successful market timing is clearly enormous; however, just as enormous is the potential penalty to unsuccessful market timing.

The best and worst possible return paths demark the feasible set of return paths for the two asset model. All possible return paths (all possible market timing paths *f*_*i*_) fall inside the envelope made by *ρ*_*b*_ and *ρ*_*w*_. As the model has all or nothing switches, the number of possible paths of length *N* is 2^*N*^. As the data set has *N* = 99, the number of possible return paths is 2^99^ ∼ 10^29^, which is large.

### The unpredictable optimal timing path

**Fig 3:**
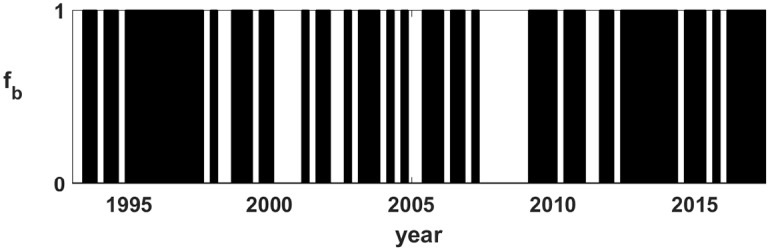
Optimal timing path. Optimal timing path *f*_*b*_ that would have produced the highest possible return path *ρ*_*b*_ over the time period. Black regions have *f*_*i*_ = 1 (stocks > bonds). White regions have *f*_*i*_ = 0 (bonds > stocks).

Fig 3 shows the historical optimal timing path *f*_*b*_ that produces the highest possible return path *ρ*_*b*_ over the time period. Black regions have *f*_*i*_ = 1 (stock return > bond return). White regions have *f*_*i*_ = 0 (bond return > stock return). It will be convenient to define *p* as the fraction of time periods in which *f* = 1, which is easily calculated by summing *f*_*b*_ and dividing by *N*. For this data *p* = *p*_*b*_ ≈ 0.64: over this time period approximately 2/3 of the time stocks returned more than bonds. While the optimal timing path *f*_*b*_ is not random like a coin flip (*p*_*b*_ ≠ 1/2), Fig 3 shows no pattern readily discernible to the eye. Is *f*_*b*_ random?

It is worth distinguishing random and unpredictable. The historically optimal timing path is not a random bit sequence because ones occur about two-thirds of the time. Nonetheless, the important question is can I predict the next element in the sequence, given knowledge of the previous elements of the sequence? How can a sequence be not random but at the same time unpredictable? Consider a 6-sided die, of which four sides have a one and two sides have a zero. For each fair roll of the die there is a two-thirds probability of a one and a one-third probability of a zero, i.e. the chance of a 1 or 0 is similar to that observed in the data. Since each fair roll of the die is independent of all rolls that have come before, there is no way to predict from the past sequence of rolls what the next roll of the die will produce. Although *p*_*b*_ is not known *a priori*—in fact *p*_*b*_ could be different over different time periods or markets—the unpredictable die analogy holds for all *p*_*b*_ by changing the number of sides to the die.

Leaving the details to S2 Appendix, I use the suit of 15 tests published by NIST [10] and designed for the purpose of verifying random number generators for cryptography, using Gerhardt’s implementation of the test suite for Mathematica [11]. While most of the NIST tests, in order to ensure an accurate test, require orders of magnitude longer bit sequences than the financial time series provides, for four of the tests the *N* = 99 bit length of *f*_*b*_ is close to the suggested minimum length. Again, leaving details to S2 Appendix, the result of those four tests is that *f*_*b*_ is random (unpredictable) at the 99% confidence level.

While the historically optimal timing sequence *f*_*b*_ is clearly special in some sense—the probability of that particular sequence to occur is 2^−99^—the question is what, if anything, distinguishes *f*_*b*_ from any other random timing paths? If we look at *f*_*b*_ and randomly generated timing paths *without knowing which is which*, can we distinguish *f*_*b*_ from the masses of possible timing paths? If *f*_*b*_ is random, as the NIST tests say it is, there is nothing to tell why it is special, which says that it is not special, that just by a 2^−99^ random chance, it was special for this time period and that, in itself, *f*_*b*_ is unpredictable, i.e. it contains *no* information about any future optimal timing path.

## Probability distribution of return paths

As the optimal timing path is indistinguishable from a random sequence, I review elementary properties of random multiplicative processes, from which it follows that the highest probability outcome of market timing is a return less than the median of the PDF of market timing returns. The return PDF is estimated by Monte Carlo sampling of random timing paths. The median of the return PDF can be directly calculated as the weighted average of the returns of the assets with the weights given by the fraction of time each asset has a higher return than the other. For the time period covered by the data the median return was close to the *f* = 0.6 balanced index fund.

### Monte carlo

**Fig 4:**
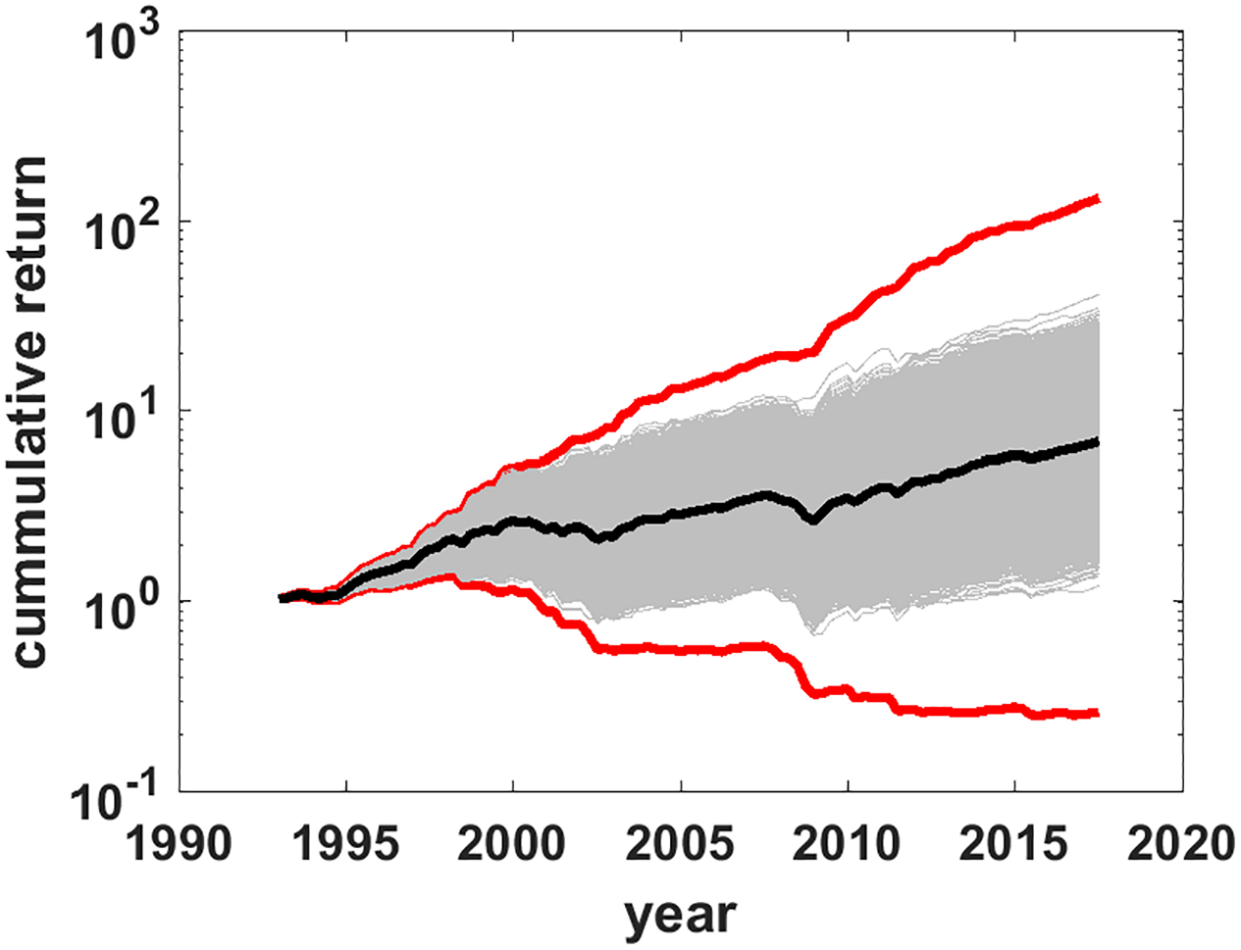
Return paths for random timing paths. Return paths (gray) for *M* = 10^5^ randomly generated timing paths. Red lines are the best and worst market timing return paths. The black line is the observed *f* = 0.6 balanced fund returns.

The distribution of typical returns of the model can be estimated by Monte Carlo methods. Generate *M* random timing paths of length *N* and calculate *M* return paths with Eq (2). In order to match the period data, set random timing paths to have the same fraction of ones and zeros as the data, i.e. the average value of *p* for the *M* timing paths is set to *p* = *p*_*b*_. This is done by using Matlab’s rand function to generate a length *N* sequence of random real numbers *n* drawn from a uniform distribution in the range [0, 1] and setting each term in the sequence equal to one if *n* < *p*_*b*_ or to zero if *n* ≥ *p*_*b*_. Fig 4 shows *M* = 10^5^ return paths as thin gray lines in a semi-log plot similar to Fig 2(c). Red lines are the boundaries of the feasible set, *ρ*_*b*_ and *ρ*_*w*_, while the thick black line is the data for the *f* = 0.6 balanced fund. Before further examination of the return PDF it will be useful to review several facts about distributions from random multiplicative processes, such as that of Eq (2).

### Random multiplicative processes

A *sum* of random numbers is guaranteed by the central limit theorem to converge to a Gaussian (normal) PDF in the limit of a large number of terms in the sum. A *product* of random numbers, such as that used in Eq (2) to calculate return, does not share this nice property. On the contrary, the PDF for a random multiplicative process (of positive numbers) depends on rare sequences that generate an asymmetric PDF with a long tail. The average value of the PDF (or of any moment) depends sensitively on the sampling size *M* and, until *M* approaches the number of possible outcomes, becomes larger and larger compared to the mode [12].

Nonetheless, what can be done is to take the log of the geometric mean of Eq (2) to change the product of returns to a sum of the log returns:

$$\begin{array}{c} \log\left(\rho_{j}^{1/N}\right)=N^{-1}\sum_{i}^{N}\log\left(f_{i}r_{si}+\left(1-f_{i}\right)r_{bi}\right). \end{array}$$ (4)

Eq (4) says that the log of the geometric mean is given by the average of the log return. The PDF of log return then does obey the central limit theorem to converge to a Gaussian PDF. Moreover, if the log of something is distributed as a Gaussian, then the something has a log-normal PDF [12]. In other words, the return PDF for market timing is log-normal, as a simple consequence of elementary properties of the logarithm. Further, if *μ* and *σ* are respectively the median and variance of the Gaussian PDF, then *e*^*μ*^ is the median and *e*^*μ* − *σ*^2^^ is the mode of the log-normal PDF: the mode, which is the most probable outcome, is less than the median of the log-normal PDF. Thus from elementary considerations the most probable outcome from market timing is a return that is less than the median of the return PDF.

**Fig 5:**
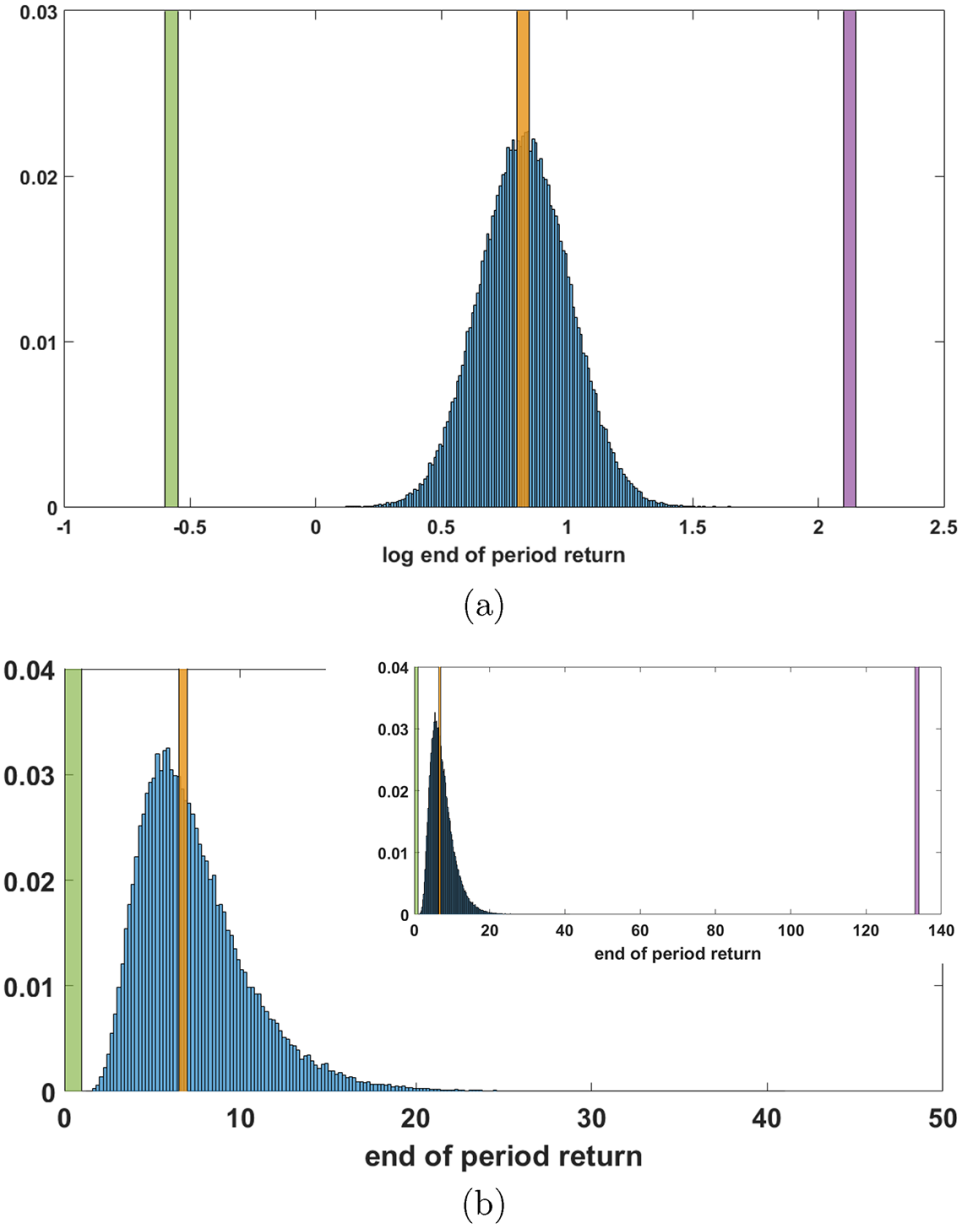
Probability distribution functions. Probability distribution function of (a) log-return and (b) return estimated from *M* = 10^5^ trials with *p* = *p*_*b*_ ≈ 0.64. Green and purple vertical bars are respectively the worst and best timing portfolios. The orange bar is the median of the PDF and the observed return of the *f* = 0.6 balanced index fund, which so closely approximates the median as to be indistinguishable at this scale. Inset of (b) is the full data range, showing the extreme low probability position of the optimum timing portfolio (purple bar) in the tail of the distribution.

To illustrate, Fig 5(a) plots the histogram of end of period log returns from the Monte Carlo data of Fig 4. Even though *M* = 10^5^ grossly under samples the order 10^29^ distinct paths in the feasible set, convergence to a Gaussian PDF is evident, as predicted by the form of Eq (4). The green and purple bars at the extremes are the results for respectively *ρ*_*w*_ and *ρ*_*b*_. The orange bar marks the median log-return and the log -return for the *f* = 0.6 balanced index fund, which are indistinguishable in this plot, and the reason for this will be discussed in the next section. Fig 5(b) plots the histogram of the end of period return (not log-return). The predicted log-normal form with a long tail is also evident. The inset shows the entire data range to indicate how long the return tail is. Colored bars have the similar meaning as in Fig 5(a), just for the return PDF instead of the log return PDF. The highest probability outcome is the mode (maximum) of the distribution, which is less than the median return marked by the orange bar.

### Expectation value of the median

The expectation value operator **E** gives the most probable value of a PDF. After a calculation given in detail in S3 Appendix, the expectation value of Eq (4) for the median *μ* of the log return distribution is

$$\begin{array}{c} \mu =E\left[\log\left(\rho_{j}^{1/N}\right)\right]=\log\left(p_{b}{\bar{r}}_{s}+\left(1-p_{b}\right){\bar{r}}_{b}\right), \end{array}$$ (5)

where ${\bar{r}}_{s,b}$ are the geometric mean returns of the stock and bond assets. Recall *p*_*b*_ is the observed fraction of time periods that the stock return exceeds the bond return. The median of the distribution of log returns is given by the log of the weighted average of the two assets with the weights given by the fraction of time periods *p* that each asset’s return exceeded that of the other. The median of the return PDF is *e*^*μ*^.

Note that because over the time period of the data *p*_*b*_ ≈ 0.64, that using the log return for the *f* = 0.6 balanced fund for the right hand side of Eq (5) well approximates the exact result for *ρ*_*b*_, which, of course, cannot be known *a priori*. As noted above, in Fig 5 the median return and the return for the *f* = 0.6 balanced index fund are indistinguishable at the scale of the plot.

It is important to note that Fig 5 shows the PDF for *costless* market timing. In practice, market timing costs higher than the index fund costs would shift the PDF to the left, but the boundaries of the feasible set and the median of the PDF would not shift because they are calculated from fund data, which already includes the small index funds costs. In practice what the Monte Carlo simulation estimates is the lower bound of the most likely shortfall of market timing to the median return given by the appropriately weighted static portfolio.

## Discussion

**Fig 6:**
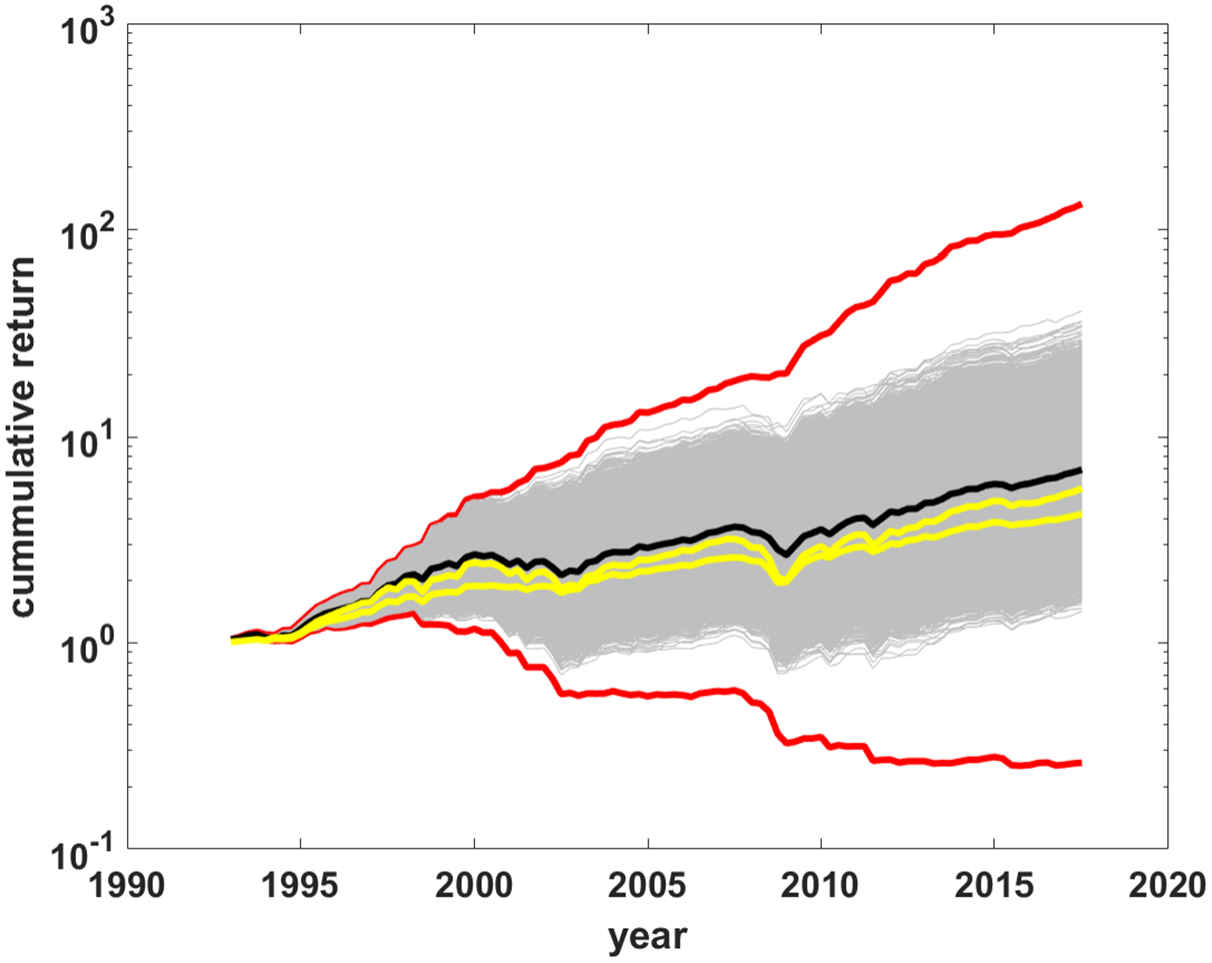
Market timing funds in the feasible set. Reprise of Fig 4 with the addition of two market timing funds with publicly available data of comparable length (yellow lines). Red lines are the best and worst timing portfolio return paths. The black line is the observed *f* = 0.6 balanced index fund.

Several critiques could be leveled at the analysis in this paper. For example, adherents of market timing would claim that their timing systems are not random, therefore they would be able to choose timing paths to have returns far out on the right tail of the PDF, i.e. that the strategy to generate random paths (random *f* sequences) is not representative of actual market timing. There are two answers to this. One is that the feasible set is well-defined and that it is simply a fact that all market timing paths, *no matter how they are generated*, are contained in the feasible set. As such, any sampling of the feasible set generates valid timing paths. The second answer is in Fig 6, which reproduces Fig 4 with the addition of the return paths (yellow lines) of two funds that Morningstar classifies as TAA funds and for which there are publicly available returns data from 1994, almost as long as for the index funds data series. S4 Appendix has details about these two funds, which are rated by Morningstar as above average. While these market timing funds were neither limited to two asset classes, nor did they make all or nothing switches, yet their return paths are, as expected, contained inside the feasible set. The conclusion is that real-life market timers are correctly characterized—except for costs—by the PDF within the feasible set, and that random sampling of the PDF does properly characterize the return distribution expected from market timing schemes.

Fig 6 also illustrates the main result with live, not simulated, market timing data. These long-lived, above average market timing funds trailed the median return over the time period—and its close proxy, the *f* = 0.6 balanced fund—as simple math says is the most probable outcome. This longer-term observation is consistent with more recent analysis covering a much shorter time period but many more TAA funds [7]. (From Ptak [7], “We found that very few tactical funds generated better risk-adjusted returns than Vanguard Balanced Index over the extended time period we studied. Not only has the group of tactical allocation funds underperformed, but not a single one of them outperformed the simple, low-cost, passive fund.”)

A more subtle criticism is that I have not disproved market timing. This is because of the possibility of hidden variables. Hidden variables represent information, such as earnings, book value, anything, that a market timer could put into a function that produces a timing path. While the observed optimal timing path *f*_*b*_ is random to the extent that it passes the NIST tests, it is possible that there was a set of hidden variables that could have been combined in a function that would have produced the optimal timing path *f*_*b*_. Good pseudo-random number generators also pass the NIST tests but are produced by deterministic systems. Taking into account the fund data of Fig 6, I think it highly unlikely, but it could be true and so market timing is not mathematically disproved. Take comfort in that, dear reader, as you will.

## Conclusion

I have examined a two asset, all or nothing market timing model with 24 years of data from US stock and bond total market index funds from 1993–2017. The model is deliberately kept simple in order to see the basic mathematics of market timing at work answering the question, what is the likelihood of successful market timing? The boundaries of the feasible set of market timing paths, within which all market timing return paths must lie, is easy in hindsight to calculate by always choosing the higher or lower returning asset each quarter. The historical optimal timing path is, however, indistinguishable from a random sequence; it is unpredictable and encodes no information about the future optimal timing path.

The key observation is that return is a multiplicative process and so its PDF is log-normal. The implication is the mathematical fact that the most probable outcome from market timing is a below median return—even before accounting for costs. This stems from an elementary property of the logarithm. Put another way, simple math says the most likely outcome of market timing is under performance. Exactly what this under performance is can be ascertained because the median of the market timing return PDF can be directly calculated as a weighted average of the returns of the model assets with weights given by the fraction of time periods each asset has a higher return than the other. For the time period of the data the median return was close to the return of the static 60:40 stock:bond balanced index; althrough, the value of *p*_*b*_ need not be fixed for all time.

For simplicity of analysis and clarity of results the model in this paper has only two asset classes; however, it is clear that the methodology could be extended to any number of asset classes.

## Supporting information

S1 File Data and Matlab code. Matlab code with all data for calculations and figures.
(M) Click here for additional data file.

S1 Appendix Index funds. Fund data scrapped from Yahoo Finance on 2 November 2017; applicable terms of service were complied with.
(PDF)

S2 Appendix NIST test suite. The NIST test suite [10] consists of 15 statistical tests that test the randomness of binary sequences.
(PDF)

S3 Appendix Calculation of expectation value. (PDF)

S4 Appendix Tactical allocation funds. Fund data and objectives summaries scrapped from Yahoo Finance 6 November 2017; applicable terms of service were complied with.
(PDF)
