# Supplementary material for: The mathematics of market timing
Source: PLoS One. 2018 Jul 18;13(7):e0200561. doi: 10.1371/journal.pone.0200561 (PMC6051602; doi:10.1371/journal.pone.0200561)
Supplement: S1 Appendix — Fund data scrapped from Yahoo Finance on 2 November 2017; applicable terms of service were complied with. (PDF) [file pone.0200561.s002.pdf]

S1 Appendix.

Index Funds. Fund data scrapped from Yahoo Finance on 2 November 2017; applicable terms of service were complied with. Data covers 1993Q1 through 2017Q3 for

Vanguard Total Stock Market Index (VTSMX)

Vanguard Total Bond Market Index (VBMFX)

Vanguard Balanced Index (VBINX)
