# Supplementary material for: The mathematics of market timing
Source: PLoS One. 2018 Jul 18;13(7):e0200561. doi: 10.1371/journal.pone.0200561 (PMC6051602; doi:10.1371/journal.pone.0200561)
Supplement: S2 Appendix — The NIST test suite [10] consists of 15 statistical tests that test the randomness of binary sequences. (PDF) [file pone.0200561.s003.pdf]

S2 Appendix.

NIST Test Suite. The NIST test suite [10] consists of 15 statistical tests that test the randomness of binary sequences. The suite is designed to test the quality of random or pseudo-random number generators, which is why for a reliable result most of the tests require much longer (by several orders of magnitude) data sequences than that available from the financial time series of stock and bond returns. However, for four of the tests a 99 bit sequence is close to the minimum suggested length of 100 bits. It should be noted that two of the 15 tests test for uniformity of the binary sequence—the equal probability of the number of ones and zeros—and so are not applicable to the historically optimal timing sequence. I use Gerhardt's implementation of the test suite for Mathematica [12].

The following, taken from [10], gives a brief description of the four NIST tests I use. After each description I give the P-value for the test, where $P \geq 0.01$ indicates f_b is random to a 99% confidence level. The four tests are:

Runs Test. The purpose of the runs test is to determine whether the number of runs of ones and zeros of various lengths is as expected for a random sequence. In particular, this test determines whether the oscillation between such zeros and ones is too fast or too slow. $P = 0.80$.

Discrete Fourier Transform (Spectral) Test. The purpose of this test is to detect periodic features (i.e., repetitive patterns that are near each other) in the tested sequence that would indicate a deviation from the assumption of randomness. $P = 0.32$.

Serial Test. The purpose of this test is to determine whether the number of occurrences of the 2^m m-bit overlapping patterns is approximately the same as would be expected for a random sequence. $P = 0.50$.

Cumulative Sums Test. The purpose of the test is to determine whether the cumulative sum of the partial sequences occurring in the tested sequence is too large or too small relative to the expected behavior of that cumulative sum for random sequences. $P = 0.01$.
