# Supplementary material for: The mathematics of market timing
Source: PLoS One. 2018 Jul 18;13(7):e0200561. doi: 10.1371/journal.pone.0200561 (PMC6051602; doi:10.1371/journal.pone.0200561)
Supplement: S3 Appendix — (PDF) [file pone.0200561.s004.pdf]

S3 Appendix.

Calculation of Expectation Value. Start with the geometric mean of eq [2], take the log of both sides then apply the expection operator to obtain

$$E\left[\log\left[\rho^{1/N}\right]\right] = E\left[\log\left[\left(\prod_{i}^{N}(f_i r_{si} + (1-f_i) r_{bi})\right)^{1/N}\right]\right]. \tag{6}$$

Denoting $\mu = E\left[\log\left[\rho^{1/N}\right]\right]$, by linearity of the expectation operator

$$\mu = N^{-1}\sum_{i}^{N} E\left[\log\left(f_i r_{si} + (1-f_i) r_{bi}\right)\right] \tag{7}$$

and by Jensen's inequality

$$\mu \leq N^{-1}\sum_{i}^{N} \log\left(E\left[f_i r_{si} + (1-f_i) r_{bi}\right]\right). \tag{8}$$

For simplicity I assume the equality holds in eq 8 in light of the supporting numerical results. Then by repeated use of the linearity of E, the expression inside the log becomes

$$\begin{aligned} E\left[f_i r_{si} + (1-f_i) r_{bi}\right] = {} & E\left[f_i\right] E\left[r_{si}\right] + \mathrm{C}ov\left[f_i, r_{si}\right] \\ & + \left(1 - E\left[f_i\right]\right) E\left[r_{bi}\right] + \mathrm{C}ov\left[1 - f_i, r_{bi}\right], \end{aligned} \tag{9}$$

where $\mathrm{C}ov$ is covariance. Since the f_i are created in this paper as random sequences, they are uncorrelated with the stock and bond returns r_{si} and r_{bi}, and both covariance terms are zero; althrough, timing paths could be constructed that are corrrelated with returns, in which case the covariance terms would be non-zero. By construction $E\left[f_i\right] = p_b$. And, after noting that $E\left[r_{si}\right] = \bar{r}_s$ and $E\left[r_{bi}\right] = \bar{r}_b$ are the geometric means of respectively the stock and bond returns, eq [5] results.
