# Supplementary material for: The mathematics of market timing
Source: PLoS One. 2018 Jul 18;13(7):e0200561. doi: 10.1371/journal.pone.0200561 (PMC6051602; doi:10.1371/journal.pone.0200561)
Supplement: S4 Appendix — Fund data and objectives summaries scrapped from Yahoo Finance 6 November 2017; applicable terms of service were complied with. (PDF) [file pone.0200561.s005.pdf]

S4 Appendix.

Tactical Allocation Funds. Fund data and objectives summaries scrapped from Yahoo Finance 6 November 2017; applicable terms of service were complied with. Data covers 1994Q1 through 2017Q3. I do not mean to pick on these funds. They were the only ones in Morningstar that were both in the tactical asset allocation category and had a sufficiently long, publicly available return series. As Morningstar rates both funds four out of five stars, and they have been in business 23 years, and they have billions of dollars of assets under management, clearly these funds are above average, successful market timing funds. Only, they did not produce above average returns for their investors.

Putnam Dynamic Asset Allocation Balanced (PABAX). The fund allocates 45% to 75% of its assets in equities and 25% to 55% in fixed income securities. It invests mainly in equity securities (growth or value stocks or both) of both U.S. and foreign companies of any size. The fund also invests in fixed-income investments, including U.S. and foreign government obligations, corporate obligations and securitized debt instruments (such as mortgage-backed investments).

Putnam Dynamic Asset Allocation Conservative (PACAX). The fund allocates 15% to 45% of its assets in equities and 55% to 85% in fixed income securities. It invests mainly in fixed-income investments, including U.S. and foreign government obligations, corporate obligations and securitized debt instruments. The fund also invests, to a lesser extent, in equity securities (growth or value stocks or both) of U.S. and foreign companies of any size.
